# Designing AI-Enabled Video Monitoring Clinician Dashboard for Neuropsychiatric Symptoms: A Survey of User Needs

**DOI:** 10.1016/j.osep.2026.01.001

**Published:** 2026-02-08

**Authors:** Christine E. Gould, Carter H. Davis, Narayan Schüz, F. Vankee Lin, Quincy M. Samus, Tracy Terada, Merryn Daniel, Silvia Tee, Ehsan Adeli

**Affiliations:** Department of Psychiatry and Behavioral Sciences, Stanford University School of Medicine, Palo Alto, CA; Geriatric Research, Education and Clinical Center (GRECC), VA Palo Alto Health Care System, Palo Alto, CA; Geriatric Research, Education and Clinical Center (GRECC), VA Palo Alto Health Care System, Palo Alto, CA; Department of Psychiatry and Behavioral Sciences, Stanford University School of Medicine, Palo Alto, CA; Department of Psychiatry and Behavioral Sciences, Stanford University School of Medicine, Palo Alto, CA; Johns Hopkins School of Nursing, Baltimore, MD; Clinical Excellence Research Center (CERC), Stanford University School of Medicine, Palo Alto, CA; Department of Psychiatry and Behavioral Sciences, Stanford University School of Medicine, Palo Alto, CA; Geriatric Medicine Section, Division of Primary Care and Population Health, Stanford University School of Medicine, Palo Alto, CA; Department of Computer Science, Stanford University, Palo Alto, CA; Stanford Institute for Human-Centered Artificial Intelligence, Stanford University, Palo Alto, CA; Department of Psychiatry and Behavioral Sciences, Stanford University School of Medicine, Palo Alto, CA

**Keywords:** Artificial intelligence, clinician dashboard, home monitoring, neuropsychiatric symptoms, survey

## Abstract

**Objective:**

This study aimed to gather input from clinicians who assess and treat neuropsychiatric symptoms (NPS) to inform the development of a clinician dashboard to accompany an AI-enabled video-based monitoring system.

**Methods:**

*The clinician survey inquired about the importance of tracking different NPS and about additional information or features desired for the dashboard. Responses (*n *= 28) were grouped into prescribing and nonprescribing clinicians for sensitivity analyses.*

**Results:**

The most important NPS to be detected were agitation/aggression, nighttime behaviors, depression, and anxiety. Multiple environmental factors were endorsed as being very important including: behavior frequency, intensity, and time of day.

**Conclusions:**

Findings demonstrate that the desired features of the dashboard were consistent across both prescribing and nonprescribing clinicians. Notably, some of the important symptoms and features that clinicians desired in a dashboard could not be extracted from existing sensor-based systems, but would be possible with an AI-enabled video monitoring system.

## INTRODUCTION

Neuropsychiatric symptoms (NPS) encompass changes in behaviors (e.g., disinhibition, aggression, agitation), affect (e.g., anxiety, depression, apathy), and thinking and perception (e.g., delusions, judgment changes). These symptoms may precede the development of mild cognitive impairment and dementia or develop concurrently with these disorders.^1^ Moreover, the presence of NPS is distressing to the individual experiencing these symptoms, can contribute to caregiver strain, and may lead to institutionalization.^2^

One challenge to treating NPS is the lack of efficient ways to objectively identify track them over time so as to measure the efficacy of nonpharmacological and pharmacological treatments. Most gold standard measures, such as the Neuropsychiatric Inventory,^3^ rely on caregiver reports which are prone to recall bias. More intensive tracking, such as behavioral monitoring logs, provides more measurement details which can guide intervention selection, but is burdensome for caregivers who are already managing multiple responsibilities. More convenient and automated tracking, such as accelerometry and other sensors, may help identify movement-based NPS such as agitation and aggression;^4^ however, monitoring systems that have emerged focus on discrete challenges such as falls or wandering rather than the entire range of possible NPS.^5^ To date, very few home-based detection systems have utilized video-based monitoring. Video-based monitoring provides rich information, but would necessitate the development of AI algorithms to identify specific NPS and related temporal and environmental patterns, similar to those developed for hospital spaces.^6^ This high-density information has the potential to help caregivers, clinicians, and patients through the development of timely and individualized treatment of NPS.

As AI models are being trained to sort through high-density clinical information, one must consider how best to present this information to the end user. Clinical “dashboards” display summaries of individual patient data and facilitate clinicians’ interpretation and use of the data. Much of the previous research on dashboards focuses on the application to health care administration or population health management, with fewer dashboards supporting direct patient care.^7^ Moreover, to our knowledge, no studies have explored clinician perspectives on dashboards designed to monitor NPS.

The aim of the present study was to gather input from clinicians who assess and treat NPS to inform the development of a clinician dashboard to accompany an AI-enabled video-based monitoring system. Additionally, we aimed to understand what NPS should be tracked and what features clinicians desired.

## METHODS

The Stanford University IRB approved this study as part of the larger study protocol focused on detecting NPS in community-dwelling older adults. The survey was distributed between December 2024 and February 2025 via REDCap links emailed to clinicians and researchers who work with older patients at risk of developing Alzheimer’s Disease and related neurodegenerative diseases associated with NPS at an academic medical center and its affiliated VA hospital. Two reminders were emailed to the clinicians. Informed consent was obtained via the REDCap form that preceded the survey. Respondents were provided with an electronic gift card.

The survey gathered information about the respondent’s discipline, settings where they deliver care, and how frequently they treat patients with NPS in their practice. Nine items assessed comfort with various aspects of assessment and management of NPS using a 5-point Likert-type scale ranging from very comfortable to very uncomfortable. Regarding the clinician dashboard, respondents were asked to rank how important each of the twelve symptoms is to capture and monitor. The items or symptoms were drawn from the categories on the Neuropsychiatric Inventory,^3^ and each item was ranked as very important, somewhat important, or less important. A free response item invited participants to provide comments about additional NPS symptoms to capture using the dashboard.

Clinicians were asked to review a list of scenarios in which they might use the dashboard to view a patient’s NPS history. These scenarios developed by the research team, which include experts in geriatric medicine, geriatric mental health, neuroscience, and computer engineering. The survey included a free response box to share additional scenarios not included on the list. Two questions ascertained whether caregivers of persons with NPS whom clinicians are seeing are currently using video-based home monitoring and for what reasons. Demographics items were also included to ascertain the race/ethnicity, gender, and age group of respondents. Frequencies, percentages, and descriptive statistics summarized survey responses.

Open-ended items were summarized using content analysis.^8^ The first and second authors created categories and coded the data. Discrepancies were adjudicated using a consensus approach.

## RESULTS

Thirty-one respondents completed the survey; however, 3 only completed partial background information and demographics, which yielded 28 respondents for most analyses. As shown in Table 1, most respondents were physicians, nurses, or psychologists. Respondents represented outpatient, inpatient, home-based primary care, and skilled nursing settings. Most worked in multiple settings (n = 12, 38.7%) or outpatient settings only (n = 11, 35.5%).

We conducted analyses to examine whether there were differences in comfort in managing NPS for prescribing and nonprescribing clinicians. Notably, comfort in screening for and assessing changes in cognitive impairment was overall high, with almost all respondents being comfortable to very comfortable with these tasks. Prescribing clinicians were more comfortable managing cognitive impairment, NPS, and late-life depression or mental health concerns using pharmacological interventions compared to clinicians without prescribing privileges (Supplementary Table 1). There were no differences between prescribing and nonprescribing clinicians in their comfort using nonpharmacological interventions to manage NPS.

The most important NPS to be detected were agitation/aggression, nighttime behaviors, depression, and anxiety (Supplementary Fig. 1). The least important behaviors were elation, followed by apathy. Key environmental factors clinicians deemed very important to identify included: frequency of the behavior (71%), intensity (67.7%), time of day (61.3%), location when the symptom was detected (54.8%), presence of others (51.6%), duration of behavior (51.6%), and presence of noise, clutter or other environmental factors (48.4%). No significant differences emerged for prescribing and nonprescribing clinicians identification for the most important environmental factors to capture using the dashboard (Supplementary Table 2).

Sixteen of 28 respondents (57.1%) provided open-ended responses to the question about what additional NPS symptoms should be included in the dashboard beyond the items listed that were derived from the Neuropsychiatric Inventory (Cummings, 2020). Responses were not related to specific symptoms that should be included but rather described additional information that could be added to the dashboard related to the NPS. These responses were categorized into 4 areas: historical information (e.g., past hospitalizations, interventions tried previously), events or activities before NPS (triggers or antecedents), events or activities after NPS (response or consequence), and other environmental factors (see Supplementary Table 3).

Respondents were asked, based on their experience, to estimate what percentage of caregivers are using home monitoring devices or surveillance devices at home to monitor their loved ones, such as outdoor cameras to detect potential wandering, indoor cameras, or other types of monitoring devices. Use was reported to be low, with 12 (38.7%) respondents reporting that 0 to 24% of caregivers were using home monitoring devices, 8 (25.8%) reporting 25 to 49% of caregivers using devices, and 2 (6.5%) reporting 50 to 75% using devices. Notably, 6 (19.4%) were unsure. Caregivers endorsed several reasons for use, with multiple responses permitted: patient supervision (n = 23; 74.2), fall detection (n = 19, 61.3%), peace of mind (n = 17, 54.8%), prevention of wandering (n=14, 45.2%) and communication with patient (n = 5, 16.1%). Approximately one third (n = 11; 35.5%) of respondents reporting recommending that caregivers use video monitoring for NPS, with no significant differences between prescribing and nonprescribing clinicians.

## DISCUSSION

This study represents a critical first step in gathering input from clinicians on dashboard features needed to detect and monitor NPS. Findings demonstrate that despite differences among clinicians’ comfort in managing NPS, the desired features remain the same for both user subgroups. Notably, some important symptoms and features that clinicians desired in a dashboard could not be extracted from existing sensor-based systems and warranted the use of video monitoring. Specifically, while the behavioral (e.g., agitation/aggression, nighttime behaviors) symptoms may be extracted using accelerometry, affective NPS (e.g., irritability, anxiety, depression) would need a different modality of monitoring. An interesting finding was that apathy was not highly ranked as being important to monitor. It is possible that apathy may not be as disruptive to care compared with more behavioral symptoms, but nevertheless, it is a prominent symptom across multiple subtypes of dementia.^1^

**FIGURE 1.**
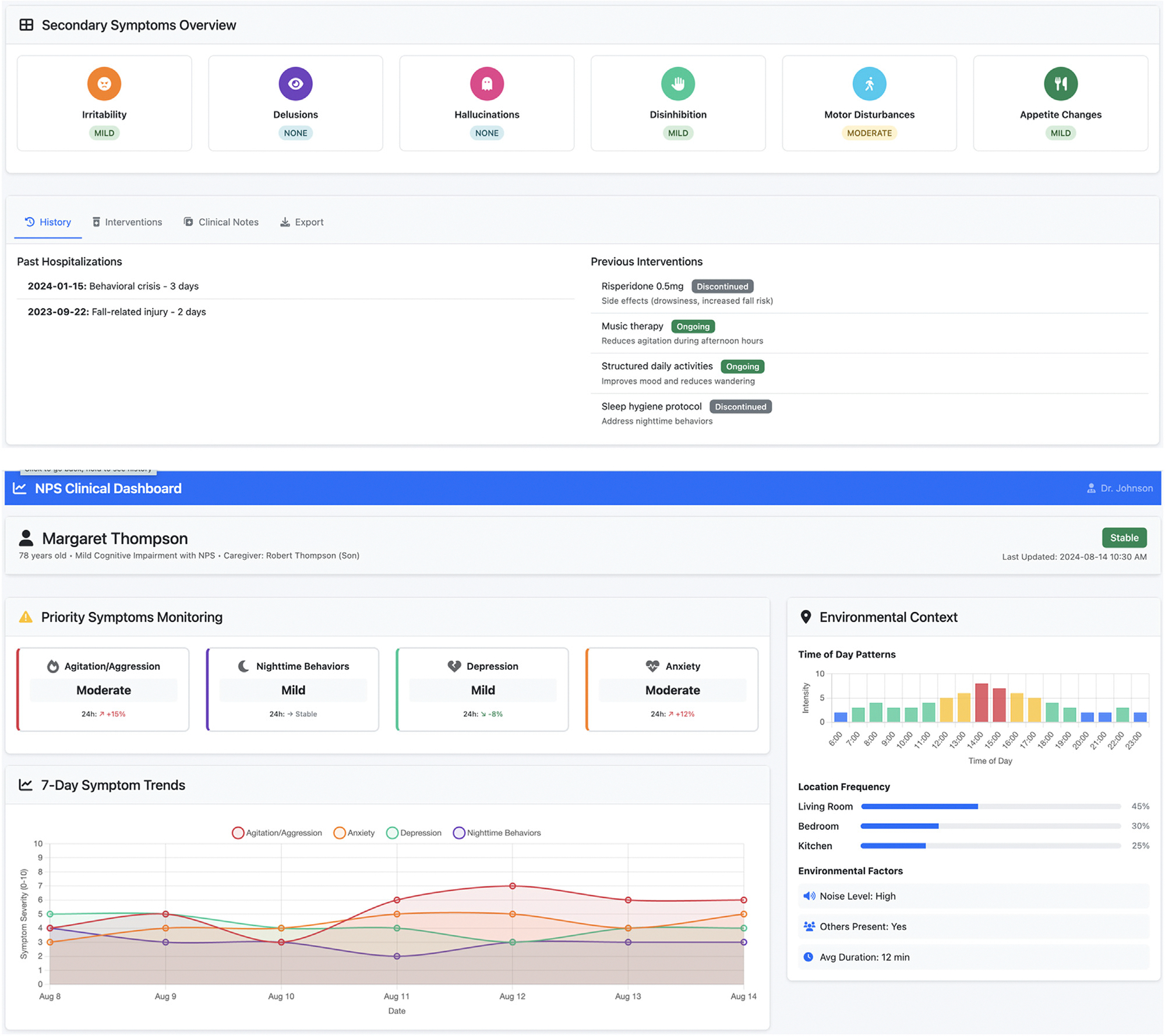
Proposed clinical dashboard.

Clinicians expressed strong interest in gathering more information about the context in which the NPS occur and the diurnal patterns of NPS. These features would necessitate the development and validation of AI algorithms to provide more complex data summaries. An example dashboard based on these findings is shown in Figure 1. Limitations of using these AI-based algorithms include the cost of running these computational models and older adults’ privacy-related concerns about the use of AI in health care.^9^

Next steps warrant continued involvement of end-users in the iterative development of a clinician dashboard for NPS through qualitative interviews and usability testing.^10^ In summary, our study demonstrates an important step in incorporating end-users (clinicians) into the design of a dashboard summarizing findings from a home monitoring system for NPS.

## Supplementary Material

Supplementary Material

SUPPLEMENTARY MATERIALS

Supplementary material associated with this article can be found in the online version at doi:10.1016/j.osep.2026.01.001.

## Figures and Tables

**TABLE 1.** Clinician Demographics (n = 28)

	Mean/Count (SD/%)

Gender^a^	
Female	21 (67.7%)
Male	5 (16.1%)
Missing/prefer not to answer	5 (16.1%)
Age^a^	
30–39	12 (38.7%)
40–49	7 (22.6%)
50–59	4 (12.9%)
60+	3 (9.7%)
Missing/prefer not to answer	5 (16.2%)
Race/ethnicity	
Asian	13 (41.9%)
Hispanic	1 (3.2%)
White	13 (41.9%)
Multiracial	1 (3.2%)
Prefer not to answer	2 (6.5%)
Clinician discipline^a^	
Physician	19 (61.3%)
Nurse (e.g., RN, CNS)	4 (12.9%)
Psychologist	4 (12.9%)
Missing/prefer not to answer	4 (12.9%)
Care delivery setting(s)^b^	
Outpatient	22 (71%)
Home based primary care	4 (12.9%)
Inpatient	12 (38.7%)
Skilled nursing facility	8 (25.8%)
Prefer not to answer	1 (3.2%)
Frequency of patients with NPS seen in practice	
Daily	10 (32.3%)
A few times a week	14 (45.2%)
A few times a month	4 (12.9%)

Notes:

a Due to missing data, n’s vary between 26 and 27 for these items

b Respondents were asked to check all that apply.

## Data Availability

A limited data set supporting this study is available upon request.

## Highlights

### • What is the primary question addressed by this paper?

This survey identified end user preferences and design input for a clinician dashboard accompanying an AI-enabled video monitoring system to detect neuropsychiatric symptoms.

### • What is the main observation of this paper?

Clinicians expressed strong interest in gathering more information about the context in which the neuropsychiatric symptoms occur and the diurnal patterns of these symptoms.

### • How can readers use this information to improve clinical care?

These findings demonstrate the level of detail that clinicians desire to provide complex data summaries of neuropsychiatric symptoms using video monitoring system data.
