# Supplementary material for: Designing AI-Enabled Video Monitoring Clinician Dashboard for Neuropsychiatric Symptoms: A Survey of User Needs
Source: Am J Geriatr Psychiatry Open Sci Educ Pract. Author manuscript; Available in PMC 2026 Apr 15. (PMC13076236; doi:10.1016/j.osep.2026.01.001)
Supplement: Supplementary Material [file NIHMS2151603-supplement-Supplementary_Material.docx]

**Supplemental Figure 1.** Neuropsychiatric Symptoms to Capture Using the Dashboard

**Supplemental Table 1.** Comfort Managing Neuropsychiatric Symptoms

|  | Total | Non-prescribing Clinician | Prescribing Clinician | Chi-square test |
| --- | --- | --- | --- | --- |
|  |  |  |  |  |
|  | n = 28 | n = 9 | n = 19 |  |
| **Screening for cognitive impairment** |  |  |  | *χ²*= 4.93, *p* = .09 |
| Very Comfortable | 19 (61.3%) | 4 (44.4%) | 15 (78.9%) |  |
| Comfortable | 8 (25.8%) | 5 (55.6%) | 3 (15.8%) |  |
| Neutral | 0 (0%) | 0 (0%) | 0 (0%) |  |
| Not Comfortable | 0 (0%) | 0 (0%) | 0 (0%) |  |
| Very Uncomfortable | 1 (3.2%) | 0 (0%) | 1 (5.3%) |  |
| **Assessing changes in cognitive impairment symptoms** |  |  |  | *χ²*= 2.05, *p* = .56 |
| Very Comfortable | 15 (48.4%) | 4 (44.4%) | 11 (57.9%) |  |
| Comfortable | 11 (35.5%) | 5 (55.6%) | 6 (31.6%) |  |
| Neutral | 1 (3.2%) | 0 (0%) | 1 (5.3%) |  |
| Not Comfortable | 0 (0%) | 0 (0%) | 0 (0%) |  |
| Very Uncomfortable | 1 (3.2%) | 0 (0%) | 1 (5.3%) |  |
| **Managing cognitive impairment using non- pharmacological interventions** |  |  |  | *χ²*= 0, *p* = 1 |
| Very Comfortable | 14 (45.2%) | 4 (44.4%) | 10 (52.6%) |  |
| Comfortable | 14 (45.2%) | 5 (55.6%) | 9 (47.4%) |  |
| Neutral | 0 (0%) | 0 (0%) | 0 (0%) |  |
| Not Comfortable | 0 (0%) | 0 (0%) | 0 (0%) |  |
| Very Uncomfortable | 0 (0%) | 0 (0%) | 0 (0%) |  |
| **Managing cognitive impairment using pharmacological interventions** |  |  |  | *χ²*= 28.0, *p* <.001 |
| Very Comfortable | 9 (29%) | 0 (0%) | 9 (47.4%) |  |
| Comfortable | 10 (32.3%) | 0 (0%) | 10 (52.6%) |  |
| Neutral | 3 (9.7%) | 3 (33.3%) | 0 (0%) |  |
| Not Comfortable | 1 (3.2%) | 1 (11.1%) | 0 (0%) |  |
| Very Uncomfortable | 0 (0%) | 0 (0%) | 0 (0%) |  |
| **Managing neuropsychiatric symptoms (including behavioral and psychological symptoms of dementia [BPSD]) using non-pharmacological interventions** |  |  |  | *χ²*= 3.07, *p* = .22 |
| Very Comfortable | 10 (32.3%) | 5 (55.6%) | 5 (26.3%) |  |
| Comfortable | 16 (51.6%) | 3 (33.3%) | 13 (68.4%) |  |
| Neutral | 2 (6.5%) | 1 (11.1%) | 1 (5.3%) |  |
| Not Comfortable | 0 (0%) | 0 (0%) | 0 (0%) |  |
| Very Uncomfortable | 0 (0%) | 0 (0%) | 0 (0%) |  |
| **Managing neuropsychiatric symptoms (including behavioral and psychological symptoms of dementia [BPSD]) using pharmacological interventions** |  |  |  | *χ²*= 18.48, *p* <.001 |
| Very Comfortable | 7 (22.6%) | 0 (0%) | 7 (36.8%) |  |
| Comfortable | 11 (35.5%) | 1 (11.1%) | 10 (52.6%) |  |
| Neutral | 3 (9.7%) | 2 (22.2%) | 1 (5.3%) |  |
| Not Comfortable | 2 (6.5%) | 1 (11.1%) | 1 (5.3%) |  |
| Very Uncomfortable | 0 (0%) | 0 (0%) | 0 (0%) |  |
| **Managing late-life depression or other mental health concerns using non-pharmacological interventions** |  |  |  | *χ²*= 2.05, *p* = .56 |
| Very Comfortable | 9 (29%) | 4 (44.4%) | 5 (26.3%) |  |
| Comfortable | 16 (51.6%) | 5 (55.6%) | 11 (57.9%) |  |
| Neutral | 2 (6.5%) | 0 (0%) | 2 (10.5%) |  |
| Not Comfortable | 1 (3.2%) | 0 (0%) | 1 (5.3%) |  |
| Very Uncomfortable | 0 (0%) | 0 (0%) | 0 (0%) |  |
| **Managing late-life depression or other mental health concerns using pharmacological interventions** |  |  |  | *χ²*= 20.36, *p* <.001 |
| Very Comfortable | 4 (12.9%) | 0 (0%) | 4 (21.1%) |  |
| Comfortable | 12 (38.7%) | 0 (0%) | 12 (63.2%) |  |
| Neutral | 4 (12.9%) | 2 (22.2%) | 2 (10.5%) |  |
| Not Comfortable | 3 (9.7%) | 2 (22.2%) | 1 (5.3%) |  |
| Very Uncomfortable | 0 (0%) | 0 (0%) | 0 (0%) |  |
| **Managing co-occurring medical conditions and geriatric syndromes such as frailty, incontinence, or frequent falls** |  |  |  | *χ²*= 4.55, *p* = .21 |
| Very Comfortable | 13 (41.9%) | 2 (22.2%) | 11 (57.9%) |  |
| Comfortable | 9 (29%) | 5 (55.6%) | 4 (21.1%) |  |
| Neutral | 5 (16.1%) | 2 (22.2%) | 3 (15.8%) |  |
| Not Comfortable | 1 (3.2%) | 0 (0%) | 1 (5.3%) |  |
| Very Uncomfortable | 0 (0%) | 0 (0%) | 0 (0%) |  |

**Supplemental Table 2.** Environmental Factors to Capture using a Dashboard

|  | Total | Non-prescribing Clinician | Prescribing Clinician | Chi-square test |
| --- | --- | --- | --- | --- |
|  | n = 28 | n = 9 | n = 19 |  |
| **Frequency** |  |  |  | *χ²*= 0.001, *p* = 1 |
| Very important | 22 (71%) | 7 (77.8%) | 15 (78.9%) |  |
| Somewhat important | 6 (19.4%) | 2 (22.2%) | 4 (21.1%) |  |
| Less important | 0 (0%) | 0 (0%) | 0 (0%) |  |
| Prefer not to answer | 0 (0%) | 0 (0%) | 0 (0%) |  |
| **Time of day** |  |  |  | *χ²*= 0.12, *p* = .73 |
| Very important | 19 (61.3%) | 7 (77.8%) | 12 (63.2%) |  |
| Somewhat important | 9 (29%) | 2 (22.2%) | 7 (36.8%) |  |
| Less important | 0 (0%) | 0 (0%) | 0 (0%) |  |
| Prefer not to answer | 0 (0%) | 0 (0%) | 0 (0%) |  |
| **Intensity** |  |  |  | *χ²*= 0.49, *p* = .78 |
| Very important | 21 (67.7%) | 7 (77.8%) | 14 (73.7%) |  |
| Somewhat important | 6 (19.4%) | 2 (22.2%) | 4 (21.1%) |  |
| Less important | 1 (3.2%) | 0 (0%) | 1 (5.3%) |  |
| Prefer not to answer | 0 (0%) | 0 (0%) | 0 (0%) |  |
| **Presence of others** |  |  |  | *χ²*= 2.61, *p* = .27 |
| Very important | 16 (51.6%) | 7 (77.8%) | 9 (47.4%) |  |
| Somewhat important | 10 (32.3%) | 2 (22.2%) | 8 (42.1%) |  |
| Less important | 2 (6.5%) | 0 (0%) | 2 (10.5%) |  |
| Prefer not to answer | 0 (0%) | 0 (0%) | 0 (0%) |  |
| **Duration** |  |  |  | *χ²*= 0.09, *p* = .77 |
| Very important | 16 (51.6%) | 6 (66.7%) | 10 (52.6%) |  |
| Somewhat important | 12 (38.7%) | 3 (33.3%) | 9 (47.4%) |  |
| Less important | 0 (0%) | 0 (0%) | 0 (0%) |  |
| Prefer not to answer | 0 (0%) | 0 (0%) | 0 (0%) |  |
| **Location when symptom was detected** |  |  |  | *χ²*= 1.99, *p* = .37 |
| Very important | 17 (54.8%) | 7 (77.8%) | 10 (52.6%) |  |
| Somewhat important | 9 (29%) | 2 (22.2%) | 7 (36.8%) |  |
| Less important | 2 (6.5%) | 0 (0%) | 2 (10.5%) |  |
| Prefer not to answer | 0 (0%) | 0 (0%) | 0 (0%) |  |
| **Noise, clutter, or other environmental factors** |  |  |  | *χ²*= 0.92, *p* = .63 |
| Very important | 15 (48.4%) | 6 (66.7%) | 9 (47.4%) |  |
| Somewhat important | 9 (29%) | 2 (22.2%) | 7 (36.8%) |  |
| Less important | 4 (12.9%) | 1 (11.1%) | 3 (15.8%) |  |
| Prefer not to answer | 0 (0%) | 0 (0%) | 0 (0%) |  |

Note. No significant differences emerged for prescribing clinician compared with non-prescribing clinician regarding their preferences for features to be included in a clinician dashboard.

**Supplemental Table 3**. Qualitative Codes, Definitions, and Counts

| **Code** | **Definition** | **Counts** | **%** | **Example Quotation** |
| --- | --- | --- | --- | --- |
| Historical information | Relevant/recent medical events such as hospitalizations, information on which interventions have been tried for NPS, how previous interventions have worked, how NPS have been reported previously (e.g., by caregiver) | 7 | 43.8% | “Historical data to use as comparison. E.g., An individual who typically eats at 6pm has shifted to an earlier feeding schedule; the typical ambient noise level in this home is 20db compared to the current 50db, etc.” |
| Additional NPS | Relevant NPS not listed in measure (e.g., NPS specific to individual patient) | 1 | 6.3% | “While there are general symptoms that are typical, there are also unique symptoms specific to that individual that should be captured on the dashboard as well” |
| Events/activities before NPS | Specific activities, interactions, or other events preceding NPS | 7 | 43.8% | “Triggers: bathing/grooming; medications administration; pain” |
| Events/activities after NPS | Specific activities, interactions, or other events following NPS | 5 | 31.3% | “what was done to MANAGE them? did it succeed? to what extent? If it did not succeed, I'd like to have place where the person can indicate why not.” |
| Other environmental factors | Stable environmental factors not specifically tied to NPS before or after, but which may influence the expression of NPS (e.g., presence of caregivers). Exclude specific activities that are limited to before or after NPS. | 3 | 18.8% | “Presence of others could also be more specific to family caregivers vs non-family caregivers to see if there are differences in behaviors.” |

Note. NPS = Neuropsychiatric Symptoms
